# How students and specialists appreciate the mini-clinical evaluation exercise (mini-CEX) in Indonesian clerkships

**DOI:** 10.1186/s12909-020-02062-z

**Published:** 2020-05-08

**Authors:** Yoyo Suhoyo, Johanna Schönrock-Adema, Ova Emilia, Jan B. M. Kuks, Janke Cohen-Schotanus

**Affiliations:** 1 Department of Medical Education, Faculty of Medicine, Public Health and Nursing, Universitas Gadjah Mada, Gd. Prof. Drs. Med. R. Radiopoetro, Lt. 6 Sayap Barat, Jl. Farmako, Sekip Utara, Yogyakarta, 55281 Indonesia; 2 Center for Education Development and Research in Health Professions, University of Groningen and University Medical Center Groningen, Groningen, The Netherlands; 3 Department of Obstetrics and Gynaecology, Faculty of Medicine, Universitas Gadjah Mada, Yogyakarta, Indonesia; 4 Institute for Medical Education, University of Groningen and University Medical Center Groningen, Groningen, The Netherlands; 5 Department of Neurology, University Medical Center Groningen, Groningen, The Netherlands

**Keywords:** Mini-CEX, Acceptability, Cultural differences, Undergraduate clerkship

## Abstract

**Background:**

Cultural differences might challenge the acceptance of the implementation of assessment formats that are developed in other countries. Acceptance of assessment formats is essential for its effectiveness; therefore, we explored the views of students and specialists on the practicality and impact on learning of these formats. This study was conducted to explore Indonesian students’ and specialists’ appreciation of the implementation of the Mini-Clinical Evaluation Exercise (Mini-CEX) in Indonesian clerkships.

**Methods:**

This study was conducted at the Universitas Gadjah Mada, Indonesia. Participants were 52 students and 21 specialists in neurology and 78 students and 50 specialists in internal medicine. They were asked to complete a 19-item questionnaire that covered the characteristics of the mini-CEX such as its practicality, and the impact on learning and professional development*.* We used a Mann-Whitney U test to analyse the data.

**Results:**

In total, 124 students (46 from neurology and 78 from internal medicine) and 38 specialists (13 from neurology and 25 from internal medicine) participated in this study.

Students and specialists were positive about the practicality of the mini-CEX and the impact of this assessment format on learning and on professional development. The Mann-Whitney U test showed that there were no significant differences between students’ and specialists’ opinions on the mini-CEX, except for 2 items: specialists’ appreciation of direct observation (mean rank = 93.16) was statistically significantly higher than students’ appreciation of it (mean rank = 77.93; z = 2.065; *p* < 0.05), but students’ appreciation of the item that students’ past mini-CEX results affected their recent mini-CEX outcomes (mean rank = 85.29) was significantly higher than specialists’ appreciation of it (mean rank = 69.12; z = 2140; *p* < 0.05).

**Conclusion:**

Students and specialists were positive about the mini-CEX in Indonesian clerkships, although it was developed and validated in another culture. We found only small differences between their appreciations, which could be explained by the patterns of specialist-student interaction in Indonesian culture as large power distance and low individualism country.

## Background

The World Federation for Medical Education (WFME) aims to promote high quality in medical education and formulated – based on expert consensus – international quality standards for medical education [1]. For medical schools, meeting these quality standards often means implementing teaching and/or assessments formats that have been developed and validated thoroughly and published and recommended in international peer-reviewed high-quality journals. An example of such an assessment format is the Mini Clinical Evaluation Exercise (mini-CEX), which was originally developed in the USA and is now being implemented around the world [2, 3]. The implementation of assessment formats from other countries is a challenge because of the differences in culture [4, 5]. Cultural differences may inhibit the acceptance of new assessment formats by important stakeholder groups like teachers or students. Acceptance of assessment formats is essential for its effectiveness [6] so cultural differences in this context should be recognized [4, 7]. To gain insight into the acceptance of new assessment formats, it is important to explore the views of stakeholders – for instance, students and specialists who function as teachers in the clinical setting – on the practicality and impact on learning of these formats [8, 9]. Therefore, in this study, we explored students’ and specialists’ appreciation of the implementation of the mini-CEX in clerkships in a culture that differs strongly from the culture in which the mini-CEX was developed.

The mini-CEX is widely used as a valid and reliable assessment method to assess clinical skills [10–14]. It was designed to evaluate the trainee’s performance with a real patient using a structured form [10]. Direct observation and structured feedback based on this observation are needed to facilitate learning during training [10–12, 15, 16]. When used regularly in the real clinical setting, the mini-CEX may stimulate students’ development of clinical skills [3, 4]. Any desirable and undesirable behaviour that may occur during student-patient interactions can be detected early through observing students. The provision of feedback shortly after the interaction enables immediate reinforcement and correction [17, 18].

The mini-CEX has been acknowledged as a practical assessment instrument [19–24]. Furthermore, it is regarded as a valuable tool to document direct supervision of clinical skills [11, 22–26], improve specialist-student relationships [22, 24], facilitate effective feedback [11, 19, 21–24, 27], and improve learning [11, 19, 22, 24, 27, 28]. The mini-CEX has also been acknowledged as a valuable tool for improving professional development [11, 20, 24].

The mini-CEX has been positively evaluated with high acceptance in countries with a culture similar to that in which the mini-CEX was developed [11, 19–24, 26–28]. These countries are classified high on individualism and low on power distance [29]. The individualism-collectivism dimension pertains to the degree to which individuals are integrated into groups and their perceived obligation to and dependence on groups, the power distance dimension pertains to how a society deals with levels of status or social power and to the degree to which less powerful members of a society accept and expect that power is distributed unequally. Since the views and values of teachers and students about teaching, learning and assessment processes have been found to differ between cultures [7, 30, 31], the implementation of educational concepts developed in one culture in a distinct culture bears the risk that the concept is not compatible with that culture, despite efforts to take culture into account. Therefore, we aimed to investigate whether an educational concept developed in the Western world can be implemented into a different culture in such a way that it is acceptable. This study focused on the acceptance of the mini-CEX in a culture, which is – in contrast to the countries from which the mini-CEX originated – classified as low on individualism and high on power distance [29].

We know that differences between countries on the cultural dimensions of individualism and power distance can be related to differences in student-teacher interactions [32, 33]. A study in Indonesia, a country characterized as low on individualism and high on power distance, showed that the implementation of the mini-CEX was a challenge [4]. Firstly, the mini-CEX focuses on *individual* feedback instead of on group feedback, which is more common in countries classified low on individualism [4]. Secondly, the mini-CEX format prescribes that *specialists* give this individual feedback, whereas in countries classified high on power distance - traditionally - residents provide most feedback in the clinical learning environment since residents are closer to students in terms of power distance than specialists are [34]. If the set-up of a new assessment format like the mini-CEX is not compatible with the culture, the risk of non-acceptance will be high, which may negatively affect the use and effectiveness of the mini-CEX [6, 7, 35]. Therefore, we decided to examine the acceptance of the mini-CEX in a substantially different culture: after a careful implementation process, we investigated the appreciation of this new assessment format by students and specialists in an Indonesian context.

## Methods

### Context

This study was performed in a culture characterized by low individualism and high power distance, which implies a strong contrast with the culture in which the mini-CEX was developed (high on individualism and low on power distance). This study was conducted at the Faculty of Medicine, Universitas Gadjah Mada, Indonesia. The duration of the medical curriculum is 5.5 years. The clinical phase consists of 2 years of department-based clerkships. The clerkship program takes place in two main teaching hospitals and several affiliated hospitals. Starting in 2009, we carefully developed and implemented the mini-CEX involving all stakeholders in the process, see Suhoyo et al. 2014 for an extensive description of the decision-making and implementation process [4]. The data collection took place between 2010 and 2012.

### The implementation of the mini-CEX

During the 11-week clerkships such as Internal Medicine, students were required to schedule at least 4 mini-CEX encounters, and during 4-week clerkships such as Neurology at least 2 mini-CEX encounters. Based on consensus between the Clinical Rotation Team (Clerkships Committee), the Education Coordinator and the Assessment Committee, students were assessed on eight clinical competencies: history taking, physical examination, diagnosis, patient management, communication/counselling, professionalism, organization/efficiency, and overall clinical care. A 4-point scale was used for scoring (1 = below expectations, 2 = meeting expectations, 3 = above expectations, and 4 = outstanding) [4]. Each student received a logbook with guidelines about the assessment process and assessment forms. All students had to bring their logbooks along during clerkships in all departments. The guidelines for the assessment process prescribe that the student asks a specialist to conduct a mini-CEX and to provide individual feedback immediately after observation. Furthermore, the guidelines for the assessment process prescribe that the specialist selects the patient for the mini-CEX. The specialists were introduced to and trained in the basic concepts of the mini-CEX (criteria and assessment procedure) and trained in providing constructive feedback. Performances on the mini-CEX were part of the final clerkship grade.

### Questionnaire

We developed a questionnaire based on existing studies that investigated trainees and specialists’ views of the mini-CEX [19, 20, 27]. This resulted in a 19-item questionnaire that covered the characteristics of the mini-CEX such as its practicality, the impact on learning, and professional development*.* The items had to be answered on a 5-point Likert scale (1 = strongly disagree, 5 = strongly agree). The questionnaire was written in Indonesian and has been applied in a pilot study among students (*n* = 32) and specialists (*n* = 15). From pilot study among students, reliability analyses indicated good internal consistency (Cronbach’s Alpha = 0.87). Subscale analysis showed relatively good internal consistency of the subscale practicality (Cronbach’s Alpha 0.69), and good internal consistencies of the subscales impact on learning and professional development (Cronbach’s Alpha 0.82 and 0.79). A pilot study among teachers, reliability analyses indicated good internal consistency (Cronbach’s Alpha = 0.84). Subscale analysis showed good internal consistency of the subscales practicality, impact on learning, and professional development (Cronbach’s Alpha 0.82, 0.88 and 0.81, respectively).

### Participants and procedure

We distributed the questionnaires to 130 students (52 students in Neurology and 78 in Internal Medicine) and 71 specialists (21 specialists working at the Neurology department and 50 working at the Internal Medicine department) to measure their experiences with the mini-CEX. The students received the questionnaire directly at the end of their clerkships in the Neurology and Internal Medicine department, respectively. After explaining the study objectives, the students completed the questionnaires and put them upside-down on a table in front of the room. Specialists received the questionnaires from supporting staff in each department with accompanying letters explaining the objectives of the study. After completion, they returned the questionnaire to the supporting staff, who passed the questionnaires on to us. Students and teachers completed the questionnaires voluntary and anonymously and did not receive any reward for their participation. We obtained ethical approval for the study from the Medical and Health Research Ethics Committee (MHREC) at Gadjah Mada University.

### Data analysis

We compared the numbers of students and specialists between both departments of Neurology and Internal Medicine using the Chi Square. To explore whether differences in responses existed between students and specialists, we compared students’ and specialists’ perceptions using the Mann-Whitney U-test. We used the Mann-Whitney U test, since the distribution of the data was not normal. The data was analysed using the Statistical Package for the Social Sciences (SPSS).

## Results

Response rates were 95% among the students (*N* = 124; 46 students from Neurology, 61% female and 39% male, and 78 from Internal Medicine, 59% female and 41% male) and 54% among the specialists (*N* = 38; 13 specialists from Neurology and 25 from Internal Medicine). We did not find significant differences between departments (*p* = 0.746).

In general, students and specialists were positive to very positive about the practicality of the mini-CEX and about the impact of this assessment format on learning and on professional development. The Mann-Whitney U test showed that there were no significant differences between students’ and specialists’ opinions on the mini-CEX, except for 2 items. Specialists considered direct observation slightly more important for judging students’ skills than students did themselves (*p* = 0.039). On the other hand, students were slightly stronger of the opinion that outcomes from previous mini-CEXs influenced their current results than teachers were (Table 1).

**Table 1:** Students’ and specialists’ perceptions of the implementation of the mini-CE

No	What is your opinion on the Mini-CEX?	-------Students (*n* = 124)---------					------Specialists (*n* = 38)---------					Students – Specialists’ comparison			
												Median of Students (IR)	Median of Specialists (IR)	Mann-Whitney U Test	
		SD (%)	D (%)	N (%)	A (%)	SA (%)	SD (%)	D (%)	N (%)	A (%)	SA (%)			*Z*	Sig.
**A**	Practicality														
1	The Mini-CEX is a practical assessment tool	0	5	11	69	15	3	0	11	74	13	4.00 (4.00–4.00)	4.00 (4.00–4.00)	−0.172	0.863
2	The Mini-CEX is easy to use for examiners to observe my performance	0	2	14	65	20	3	8	8	68	13	4.00 (4.00–4.00)	4.00 (4.00–4.00)	−1.078	0.281
3	When assessing clinical skills, the direct observations are useful for assessing my clinical skills	0	2	5	66	27	3	0	0	53	45	4.00 (4.00–5.00)	4.00 (4.00–5.00)	−2.065	0.039*
4	The Mini-CEX forms are clear	2	3	18	65	12	0	3	24	58	16	4.00 (4.00–4.00)	4.00 (3.00–4.00)	−0.032	0.974
5	The Mini-CEX forms offer sufficient space for feedback	0	4	19	65	13	0	3	16	71	11	4.00 (4.00–4.00)	4.00 (4.00–4.00)	−0.214	0.830
**B**	**Impact on learning**														
1	The Mini-CEX stimulates clinical teachers to observe students’ interactions with patients	1	0	5	80	15	3	0	8	58	32	4.00 (4.00–4.00)	4.00 (4.00–5.00)	−1.455	0.146
2	Direct observation is a strength of the Mini-CEX	1	0	6	68	26	3	3	3	58	34	4.00 (4.00–5.00)	4.00 (4.00–5.00)	−0.728	0.466
3	The Mini-CEX has a positive effect on the student-teacher relationship	1	1	14	68	17	3	3	21	55	18	4.00 (4.00–4.00)	4.00 (3.00–4.00)	−0.907	0.365
4	The Mini-CEX has impact on students’ learning processes	1	1	8	67	23	3	3	13	68	13	4.00 (4.00–4.00)	4.00 (4.00–4.00)	−1.817	0.069
5	The Mini-CEX helps students prepare for the assessment in the final week of a clerkship	1	0	8	65	26	5	5	8	53	29	4.00 (4.00–5.00)	4.00 (4.00–5.00)	−0.538	0.590
6	The assessor’s feedback helps students to improve their weaknesses	1	1	7	62	29	0	5	11	63	21	4.00 (4.00–5.00)	4.00 (4.00–4.00)	−1.340	0.180
7	The assessor’s feedback is helpful in daily clinical practice	2	1	10	60	27	0	5	16	61	18	4.00 (4.00–5.00)	4.00 (4.00–4.00)	−1.408	0.159
8	Feedback is a strength of the mini-CEX	1	0	10	54	35	0	5	16	53	26	4.00 (4.00–5.00)	4.00 (4.00–5.00)	−1.593	0.111
9	The Mini-CEX impacts on students’ self-directed learning	1	1	11	65	22	3	8	11	58	21	4.00 (4.00–4.00)	4.00 (4.00–4.00)	−0.869	0.385
10	Students’ past Mini-CEX results affected their recent Mini-CEX outcomes	1	2	13	65	19	3	5	26	53	13	4.00 (4.00–4.00)	4.00 (3.00–4.00)	−2.140	0.032*
11	Experiences students gained from Mini-CEX assessments are applicable to daily clinical practice	1	1	8	68	23	0	5	8	63	24	4.00 (4.00–4.00)	4.00 (4.00–4.25)	−0.219	0.827
**C**	**Professional development**														
1	The Mini-CEX has influenced students’ professional development as a doctor	1	0	7	73	19	3	3	11	66	18	4.00 (4.00–4.00)	4.00 (4.00–4.00)	−0.861	0.389
2	The Mini-CEX has influenced students’ perspective on patient care	1	0	11	69	19	3	3	13	63	18	4.00 (4.00–4.00)	4.00 (4.00–4.00)	−0.718	0.473
3	The Mini-CEX has influenced students’ interactions with patients and their families	1	2	15	64	18	3	3	18	55	21	4.00 (4.00–4.00)	4.00 (3.75–4.00)	−0.191	0.848

Note: *SD* Strongly Disagree, *D* Disagree, *N* Neither agree or disagree, *A* Agree, *SA* Strongly Agree, *IR* Interquartile Range; *p < .05

## Discussion

The aim of our study was to investigate how students and specialists as clinical teachers appreciate the mini-CEX in Indonesian clerkships, and whether there are differences between them. In general, students and specialists appreciated the practicality and were positive about the general impact of the mini-CEX on learning and about its impact on professional development. We found no significant differences between students’ and specialists’ appreciation, except that specialists were significantly more positive about the topic ‘direct observation’ and students were more convinced that ‘past Mini-CEX results affected recent mini-CEX outcomes’.

The positive evaluation of the mini-CEX showed that students and specialists accepted the mini-CEX even though we implemented the educational concept of the mini-CEX in a culture substantially different from the one in which it was developed. This finding strengthened the result of our previous study that managing the implementation process carefully and taking culture and local context into account can facilitate the acceptance of the mini-CEX [4]. It showed that we can overcome the challenge raised by cultural differences. We can provide more individual feedback from specialists where traditionally students receive most individual feedback from residents instead of specialists [34]. The careful implementation of the mini-CEX in an existing program may have positively influenced the appreciation of its practicality. The positive appreciation of the mini-CEX may also have been influenced by the fact that in the Internal Medicine Department, the students who were assessed with the mini-CEX showed significantly more improvement between the first end subsequent assessments than the students who completed the clerkships before the implementation of the mini-CEX [4].

We found a significant, though small difference between students and specialists with respect to their appreciation of ‘observation’ with specialists being somewhat more convinced of its importance than students were. This outcome may be related to the fact that Indonesia is a country classified low on the dimension of individualism [29, 32]. In collectivist cultures, specialists, as clinical teachers, need frequent observation to identify students’ deviations from the group standards to maintain harmony and integration in the group. However, because in this culture specialists will deal with students as a group, observing students is usually done in front of other students, and the result of observation is used as based to provide feedback to the group [36]. So, for students, being observed may be a straining experience because they are afraid of failing and losing face. Although in the mini-CEX students were observed individually, they might still feel insecure and need time to get used to being observed individually by specialists. It might be the reason why students– compared to specialists – appreciate observation as less important for assessing clinical skills. Another explanation for the finding that specialists rated the importance of observation higher than students may be that the mini-CEX format helped specialists to focus their attention on specific aspect, thus facilitating observation.

We also found that students rated the effect of past Mini-CEX experiences on their subsequent mini-CEX outcomes higher than teachers did. This finding might be explained from the fact that the Indonesian culture is characterized by large power distance and low individualism [29, 32]. In a large-power-distance country, students need to follow the directions of the teacher, in this case the specialist. Furthermore, in a country low in individualism, meeting the expectations of teachers is an important motivating factor in student learning [37]. With the implementation of the mini-CEX, specialists have to provide both oral and written feedback on the student’s performance immediately after direct observation [4]. Students might use the individual feedback from the specialist in their past mini-CEX to set objectives for their subsequent mini-CEX. So, for students, the experience that they had in past mini-CEX may really have affected their subsequent Mini-CEX outcomes. Specialists, who are superior in large power distance cultures [29, 32], do not know the results of their students on former tests since each mini-CEX was assessed by another specialist and, hence, judge the performance of students in the mini-CEX independently. Consequently, specialists may not have been aware of the degree to which students’ previous performance may have affected their subsequent mini-CEX performance and, therefore, they may have underestimated the degree to which students’ past mini-CEX results affected their subsequent mini-CEX outcomes.

This study has some limitations. First, our study was limited to only one medical school in Indonesia, since this was the first medical school at which the mini-CEX was implemented. We did perform our study at two departments, namely Internal Medicine and Neurology, but were not able to include more departments, because these two were the only two departments at which the mini-CEX was implemented [4]. Since we were bound to limit our study to one medical school, we were not able to identify differences attributable to cultural climate within organisations and differences between regions within a country [38]. However, cultural differences between countries are in general larger than those between subcultures within countries [39]. Therefore, our findings may be generalizable to other Indonesian medical schools. We suggest that upon implementation of the mini-CEX in other institutions and departments, replication studies are needed to corroborate our findings. Second, although we carefully designed our questionnaire and piloted it, we did not search for a factor structure in our questionnaire. However, we did not intend to develop an extensively validated instrument with scales to measure different concepts, but we rather tried to include all relevant aspects that should be evaluated, since – as Schuwirth (2009) [40] indicates – the value of our evaluation questionnaire is not in the internal structure of the instrument or in its construct validity, but in the relevance of the individual items. Stated differently, each aspect included in the evaluation is intended to be taken as meaningful in itself. In line with this view, we tried to include all aspects in our evaluation questionnaire that seemed relevant for our purpose and to systematically focus our questions on and cover all relevant characteristics of the mini-CEX.

Our study indicates that it is feasible to implement educational concepts originating from a different culture in an acceptable way in another culture if the implementation process is performed in a conscientious way, taking into account cultural differences. Further research is needed to evaluate students’ and specialists’ appreciation of educational concepts such as the mini-CEX that were developed in a different culture in different cultural contexts. To be able to investigate this appreciation or acceptability, also replication of our previous study is needed, in which we implemented the mini-CEX in a very conscientious way in our context, taking into account our specific culture. Future replication studies might not only focus on contexts comparable to ours in terms of educational concept and culture, but also on contexts that differ with respect to educational concept and/or culture. A comparable context and implementation approach might be applied to validate our specific findings in our specific culture and with this particular educational concept; distinct types of contexts, in terms of culture and educational concept originally developed in Western countries to be applied in that specific culture, are needed to validate our implementation approach.

## Conclusion

In conclusion, students and specialists highly appreciated the mini-CEX in Indonesian clerkships even though the concept was developed and validated in another culture. These outcomes indicate that it is feasible to implement educational formats in an acceptable way in different cultures if culture is taken into account during the implementation process. We found only small differences between students and specialists, which could be explained by Indonesian culture. We invite medical schools from other cultures to evaluate their students’ and specialists’ appreciation of educational concepts such as the mini-CEX that were developed in a different culture, to get a better understanding of the influence of culture on globally implemented educational concepts.

## Supplementary information

**Additional file 1.** Questionnaire.

## Data Availability

Data used in this study are available from the corresponding author on reasonable request.

## Abbreviations

Mini-CEX: Mini Clinical Evaluation Exercise
