# Supplementary material for: How students and specialists appreciate the mini-clinical evaluation exercise (mini-CEX) in Indonesian clerkships
Source: BMC Med Educ. 2020 May 8;20:144. doi: 10.1186/s12909-020-02062-z (PMC7206730; doi:10.1186/s12909-020-02062-z)
Supplement: Supplementary file 1 — Additional file 1. Questionnaire. [file 12909_2020_2062_MOESM1_ESM.docx]

The questionnaire of the student perception of mini-CEX

| **No** | **What is your opinion on the Mini-CEX?** | Strongly disagree | Disagree | Neither agree nor disagree | Agree | Strongly  Agree |
| --- | --- | --- | --- | --- | --- | --- |
| **A** | **Practicality** |  |  |  |  |  |
| 1 | The Mini-CEX is a practical assessment tool |  |  |  |  |  |
| 2 | The Mini-CEX is easy to use for examiners to observe my performance |  |  |  |  |  |
| 3 | When assessing clinical skills, the direct observations are useful for assessing my clinical skills |  |  |  |  |  |
| 4 | The Mini-CEX forms are clear |  |  |  |  |  |
| 5 | The Mini-CEX forms offer sufficient space for feedback |  |  |  |  |  |
| **B** | **Impact on learning** |  |  |  |  |  |
| 1 | The Mini-CEX stimulates clinical teachers to observe my interactions with patient |  |  |  |  |  |
| 2 | Direct observation is a strength of the Mini-CEX |  |  |  |  |  |
| 3 | The Mini-CEX has a positive effect on the student-teacher relationship |  |  |  |  |  |
| 4 | The Mini-CEX has impact on my learning processes |  |  |  |  |  |
| 5 | The Mini-CEX helps me to prepare the assessment in the final week of a clerkship |  |  |  |  |  |
| 6 | The assessor’s feedback helps me to improve my weaknesses |  |  |  |  |  |
| 7 | The assessor’s feedback helps me in daily clinical practice |  |  |  |  |  |
| 8 | Feedback is a strength of the mini-CEX |  |  |  |  |  |
| 9 | The Mini-CEX impacts on my self-directed learning |  |  |  |  |  |
| 10 | My past Mini-CEX results affected my recent Mini-CEX outcomes |  |  |  |  |  |
| 11 | Experiences I gained from Mini-CEX assessments are applicable to daily clinical practice |  |  |  |  |  |
| **C** | **Professional development** |  |  |  |  |  |
| 1 | The Mini-CEX has influenced my professional development as a doctor |  |  |  |  |  |
| 2 | The Mini-CEX has influenced my perspective on patient care |  |  |  |  |  |
| 3 | The Mini-CEX has influenced my interactions with patients and their families |  |  |  |  |  |
